# Deficiency of vitamins C and E in women of childbearing age in Brazil: a systematic review and meta-analysis

**DOI:** 10.1590/1516-3180.2020.0799.R1.0904221

**Published:** 2021-03-18

**Authors:** Rosa Camila Lucchetta, Sophia de Andrade Cavicchioli, Ana Luísa Rodriguez Gini, Marcela Forgerini, Fabiana Rossi Varallo, Mariane Nunes de Nadai, Fernando Fernandez-Llimos, Patricia de Carvalho Mastroianni

**Affiliations:** I PhD. Pharmacist and Postdoctoral Researcher, Department of Drugs and Medicines, School of Pharmaceutical Sciences, Universidade Estadual Paulista (UNESP), São Paulo (SP), Brazil.; II Undergraduate Student, Universidade de Araraquara (UNIARA), Araraquara (SP), Brazil.; III Undergraduate Student, Department of Drugs and Medicines, School of Pharmaceutical Sciences, Universidade Estadual Paulista (UNESP), São Paulo (SP), Brazil.; IV Pharmacist and Doctoral Student, Department of Drugs and Medicines, School of Pharmaceutical Sciences, Universidade Estadual Paulista (UNESP), São Paulo (SP), Brazil.; V PhD. Pharmacist and Professor, Department of Pharmaceutical Sciences, Faculdade de Ciências Farmacêuticas de Ribeirão Preto, Universidade de São Paulo (FCFRP-USP), Ribeirão Preto (SP), Brazil.; VI MD, PhD. Professor, Faculdade de Odontologia de Bauru, Universidade de São Paulo (FOB-USP), Bauru (SP), Brazil.; VII PhD. Pharmacist and Professor, CINTESIS - Center for Health Technology and Services Research, Laboratory of Pharmacology, Department of Drug Sciences, Faculty of Pharmacy, Universidade do Porto, Porto, Portugal.; VIII PhD. Pharmacist and Professor, Department of Drugs and Medicines, School of Pharmaceutical Sciences, Universidade Estadual Paulista (UNESP), São Paulo (SP), Brazil.

**Keywords:** Alpha-tocopherol, Ascorbic acid deficiency, Women’s health, Cross-sectional studies, Deficiency diseases, Nutrition surveys, Vitamin E deficiency, Hypovitaminosis, Lactating, Nutritional epidemiology, Maternal nutrition

## Abstract

**BACKGROUND:**

Despite the several options available for supplements containing vitamins C and E, evidence regarding the prevalence of deficiency or insufficiency of these vitamins is weak.

**OBJECTIVES:**

To estimate the prevalence of deficiency or insufficiency of vitamins C and E and associated factors among women of childbearing age, in Brazil.

**DESIGN AND SETTING:**

Systematic review and meta-analysis conducted at a Brazilian public university.

**METHODS:**

A search from index inception until May 2020 was conducted. Meta-analyses were performed using inverse variance for fixed models, with summary proportions calculation using Freeman-Tukey double arcsine (base case). Reporting and methodological quality were assessed using the Joanna Briggs Institute tool for prevalence studies.

**RESULTS:**

Our review identified 12 studies, comprising 1,316 participants, especially breastfeeding women. There was at least one quality weakness in all studies, mainly regarding sampling method (i.e. convenience sampling) and small sample size. The prevalence of vitamin C deficiency ranged from 0% to 40%. Only vitamin E deficiency was synthetized in meta-analyses, with mean prevalences of 6% regardless of the alpha-tocopherol cutoff in plasma, and 5% and 16% for cutoffs of < 1.6-12.0 mmol/l and < 16.2 mmol/l, respectively. The cumulative meta-analysis suggested that a trend to lower prevalence of vitamin E deficiency occurred in recent studies.

**CONCLUSIONS:**

Although the studies identified in this systematic review had poor methodological and reporting quality, mild-moderate vitamin C and E deficiencies were identified, especially in breastfeeding women. Thus, designing and implementing policies does not seem to be a priority, because the need has not been properly dimensioned among women of childbearing age in Brazil.

**REGISTRATION NUMBER IN PROSPERO:**

CRD42020221605.

## INTRODUCTION

Vitamins C and E are antioxidants agents and cofactors of various metabolic and enzymatic reactions. They also participate in several physiological processes, such as in repairing oxidative stress, antitumor processes and hormone production.^1^^,^^2^^,^^3^ Vitamins C and E also participate in human embryological development and their deficiency is associated with low weight in babies, prematurity, and malformations.^4^^,^^5^

Vitamin C and E deficiencies have also been correlated with occurrences of scurvy, anemia, hyperbilirubinemia, tiredness and depressive conditions.^6^^,^^7^ These deficiencies may occur because vitamins are not synthesized in the body, but are obtained through ingestion of vegetables, legumes, fruits and nuts, among other foods.^8^^,^^9^

Despite the recognized importance of these vitamins and the known risk of vitamin deficiencies, evidence among women of childbearing age is limited. The prevalence of vitamin C deficiency in adult women has been estimated to range from 6.9% in the United States to 14% in England,^10^ while vitamin E deficiency has been found to range from 20% to 90%, depending on the population subgroup analyzed, the comorbidities presented and age.^2^ Some Brazilian primary studies have estimated that the prevalences of ascorbic acid^11^^,^^12^ and alpha-tocopherol^13^^,^^14^ deficiencies/insufficiencies are 30.8% and 62%-88.1% respectively, and have hypothesized that a problem of regional relevance may exist.

Studies eliciting the prevalence of a condition reflect the burden of this condition on society, and they assist in defining priorities for healthcare policies and decision-making. Well-designed cross-sectional studies are the most appropriate study design for estimating prevalence. If conducting these primary studies is not feasible, a systematic review gathering together the existing data may be the most appropriate approach for providing an idea of the magnitude of the problem and for achieving greater national representativeness.^15^

To our knowledge, no systematic review on this topic considering Brazilian data exists.

## OBJECTIVE

The aim of this study was to estimate the prevalences of vitamins C and E deficiencies or insufficiencies and their associated factors, among women of childbearing age in Brazil.

## METHODS

### Study design, protocol and registration

A systematic review was performed in accordance with the recommendations of the Cochrane Collaboration,^16^ Meta-analysis of Observational Studies in Epidemiology (MOOSE)^17^ and Joanna Briggs Institute (JBI).^18^ The results were reported in accordance with the Preferred Reporting Items for Systematic Reviews and Meta-Analyses (PRISMA).^19^ The protocol for this review is available at OSF^20^ and PROSPERO (CRD42020221605). This study forms part of a larger study that evaluated vitamin A, B, C, D and E, calcium, iodine, iron and zinc deficiencies in women of childbearing age in Brazil.

### Information sources, search strategy and eligibility criteria

Electronic searches were conducted through a pre-defined search strategy, which was described in the protocol,^20^ in the following databases: PubMed, LILACS, WHO, CAPES dissertations and theses (gray literature) and Scopus.^21^ The search encompassed the entire period from database inception to May 2020. The reference lists of reviews and studies that were included were also searched.

Studies that fulfilled the following inclusion criteria, in accordance with the CoCoPop acronym,^22^ were included: i) Condition: vitamin C and E deficiency or insufficiency; ii) Context: Brazil without restriction of setting; and iii) Population: women of childbearing age (15 to 49 years old) without any restriction on diseases or physiological status (i.e. non-pregnant, pregnant or breastfeeding). Data from studies that reported the deficiencies of interest using a different population classification (e.g. women aged 15 to 44 or pregnant teenagers) or different laboratory parameters, were separated for appropriate subgroup analyses.

Although cross-sectional studies are the ideal and are the study design most used for reporting prevalence, many studies have the potential to report this parameter, such as national surveys or longitudinal studies. Thus, all types of studies were included, except reviews, letters, comments, reports and case series. No language restriction was applied.

### Study selection and data extraction

Two researchers independently screened the titles and abstracts and evaluated the full-text articles. Discrepancies were resolved through consensus meetings, using another researcher as a referee. The eligibility process was conducted using spreadsheets.

The following data were independently extracted by five researchers: (i) study characteristics (type of study, analysis period and state), characteristic of the population (e.g. pregnant women), micronutrient deficiency, sampling method and funding; (ii) participants’ characteristics, i.e. ethnicity, comorbidities, drug therapy or supplement in use, body mass index (BMI), age, education and per capita income; and (iii) prevalence estimates (n/N (%)) for the total population and for subgroups when available.

### Methodological quality in individual studies

Given that no validated tool for assessing the risk of bias in prevalence studies exists, an assessment of the methodological and reporting quality based on the JBI Critical Appraisal Checklist for studies reporting prevalence data^27^ was conducted. The methodological rigor and completeness of the most critical domains were considered.^28^ The evaluation was performed independently by two reviewers. In the absence of consensus, points of disagreement were resolved by seeking the opinion of another investigator.

### Synthesis of results

Although no predefined cutoffs for assessing deficiencies of vitamins C and E were considered as inclusion criteria in the present review, only studies that considered the same cutoff were grouped.

The data synthesis was primarily done through meta-analysis. Transitivity assessment was performed by comparing CoCoPop acronyms^22^ between studies (population inclusion and exclusion criteria and subpopulation definitions). If important discrepancies were identified, sensitivity analyses with the exclusion of the study in question were performed. To conduct direct meta-analyses, the data collected were transferred and analyzed separately in the R software, version 3.6.3 (R studio 1.2.5033),^23^ using the READR^24^ and META packages.^25^

Direct proportional meta-analyses were conducted using the inverse variance method (base case) and GLMM method (sensitivity analysis).^25^ To calculate weighted summary proportions, Freeman-Tukey double arcsine transformation (PFT) (base case) and Logit transformation (PLOGIT) (sensitivity analysis) were considered in fixed-effect models (base case) and random-effect models (sensitivity analysis).^19^^,^^25^ Although high heterogeneity was expected and, therefore, a random-effect model could be expected, it has been recommended that a fixed-effect model is preferable for assessing prevalence, because otherwise the weighting will not properly consider the weights of the studies.^26^ Thus, analyses were conducted using both models, and potential differences were discussed.

The results from the meta-analysis were given as the proportion combined with its 95% confidence interval (CI), along with a list of the proportions (presented as percentages) with their respective 95% CI that had been found in the individual studies included in the meta-analysis. A Higgins inconsistency test (I^2^) with an estimator for tau^2^ was used through the DerSimonian-Laird method (base case), with statistical adjustment by means of Hartung and Knapp to a random model (sensitivity analysis).

A cumulative meta-analysis was carried out to assess changes and trends over time and to highlight emerging or decreasing conditions, along with their potential relationship with public policies that had been implemented.

Sensitivity analyses were performed by means of the leave-one-out method. Subgroup and meta-regression analyses, considering the period of analysis, state and region of Brazil, comorbidities, age or status (i.e. non-pregnant, pregnant or breastfeeding) were planned for meta-analyses with at least 10 studies. Alternative statistical methods were also conducted to validate the conclusions. Potential publication bias was assessed using rank tests (base case) and linear regression or the method of moments (sensitivity analysis), with at least 10 studies per meta-analysis.^25^

### Data sharing and data accessibility

The data that support the findings of this study are openly available in OSF at http://doi.org/10.17605/OSF.IO/J9QMH.^20^

## RESULTS

### Selection process

Our systematic review identified 1,977 records in the electronic databases after removal of duplicates (PubMed, LILACS and Scopus) and 91 additional records identified through other sources (manual search, WHO database and CAPES database of dissertations and theses). Through the selection process, 259 published papers were included in the systematic review regardless of the micronutrient assessed. These included 12 studies (14 papers) about vitamins C and E (see supplementary data: Figure S1 and Table S2, available in OSF: https://osf.io/j9qmh/),^20^ consisting of eight cross-sectional studies, three prospective cohorts and one randomized clinical trial (Table 1).

**Table 1.** Description of the characteristics of the studies included and participants

Study	Study type	Vitamins	Inclusion period	State/region	Setting	Funding	Characteristic (n)	Comorbidities	Mean BMI, kg/m^2^ (± SD)	Mean age, years (± SD)
Madruga de Oliveira A et al.^11^^,^^32^	CS	C	2002	SP/southeast	Maternity	CNPq	Pregnant smokers (40); pregnant nonsmokers (87); and breastfeeding (117)	Healthy	NR	NR/20-34 years (77.0%)
Machado et al.^12^	CS	C and E	2010-2011	SP/southeast	Outpatient	UNIFESP	Pregnant (49)	HIV+	NR	30.0 (6.5)
de Azeredo and Trugo^14^	CS	E	NR	RJ/southeast	Hospital	CNPq, FAPERJ, and CAPES	Breastfeeding teenagers (72)	Healthy	23.1 (3.2)	16.9 (1.4)
Clemente et al.^31^	RCT	E	2012-2013	RN/northeast	Maternity hospital	CNPq	Breastfeeding (109)	Healthy	NR	24.1 (5.6)
de Lira et al.^37^^,^^39^	CS	E	2010	RN/northeast	Hospital	CNPq	Breastfeeding (103)	NR (some chronic and infectious diseases were excluded)	NR	24.0 (7.0)/14 to 41 years
Garcia et al.^38^	CS	E	2008	RN/northeast	Maternity hospital	CNPq	Breastfeeding (32)	NR (some chronic diseases were excluded)	NR	25.0 (6.3)/14 to 36 years
Gurgel et al.^29^	CS	E	2009-2011	RN/northeast	Maternity hospital	UFRN	Breastfeeding (209)	Healthy	NR	NR/14 to 45 years
Monteiro et al.^30^	PC	E	NR	SP and RJ/southeast	NR	NICHD	Breastfeeding (97)	HIV+	NR	NR/20-29 years (51.5%)
da Silva Ribeiro et al.^33^	CS	E	2012-2013	RN/northeast	Hospital	UFRN	Breastfeeding (58)	NR (some chronic and infectious diseases were excluded)	NR/around 28.0	NR/around 24.0
Ribeiro et al.^34^	CS	E	2013-2014	RN/northeast	Hospital	UFRN	Pregnant (103)	NR (some chronic and infectious diseases were excluded)	NR	NR/18-24 years (59.0%)
Rodrigues^36^	PC	E	2012-2015	RN/northeast	Maternity hospital	UFRN	Breastfeeding (mothers of children born preterm and at term) (124)	NR	Mothers of preterm: 28.3 (5.2); and mothers of term: 28.3 (4.3)	Mothers of preterm: 26.0 (6.7); and mothers of term: 24.5 (6.0)
da Silva et al.^35^	PC	E	2016-2017	RN/northeast	Outpatient	UFRN	Breastfeeding (nonsmokers) (116)	NR (some infectious diseases were excluded)	NR	27.8 (7.4)

BMI = body mass index; CAPES = Coordenação de Aperfeiçoamento de Pessoal de Nível Superior; CNPq = Conselho Nacional de Desenvolvimento Científico e Tecnológico; CS = cross-sectional; FAPERJ = Fundação de Amparo à Pesquisa do Estado do Rio de Janeiro; HIV+ = human immunodeficiency virus-positive; NICHD = National Institute of Child Health and Human Development; NR = not reported; PC = prospective cohort; RCT = randomized clinical trial; RJ = Rio de Janeiro; RN = Rio Grande do Norte; SD = standard deviation; SP = São Paulo; UFRN = Universidade Federal do Rio Grande do Norte; UNIFESP = Universidade Federal de São Paulo.

### Characteristics of studies and participants

The studies were conducted between 2002 and 2017, in cities in the northeastern region (n = 8) and southeastern region (n = 4), among women who were selected mainly from maternity clinics (n = 5) and hospitals (n = 4). Only Gurgel et al.^29^ reported that they used convenience sampling, while the sampling method was not reported in the remaining 11 studies. This lack of information may point towards use of convenience sampling. All the studies received some funding (Table 1).

A total of 1,316 participants were included, and the majority were breastfeeding (n = 1,037), with mean ages in the different studies ranging from 16.9 to 30.0 years. Most of the studies included healthy women or excluded women with chronic or infectious diseases (n = 9), and also excluded participants using supplements containing vitamin C or E (n = 9). No study reported prevalence among non-pregnant and non-breastfeeding women. Gurgel et al.^29^ did not report on the use of medicines or supplements and Monteiro et al.^30^ only reported on the use of antiretroviral therapy. Furthermore, most of the studies did not report mean BMI, ethnicity, educational level or per capita income. Only five studies^11^^,^^31^^,^^32^^,^^33^^,^^34^^,^^35^ reported on the participants’ educational level, and showed that the majority had low levels (data not shown). Six studies^11^^,^^12^^,^^32^^,^^33^^,^^34^^,^^35^^,^^36^ reported on per capita income and showed that the majority of the participants had monthly per capita income of less than one minimum wage (data not shown). The main characteristics of the participants are described in Table 1.

### Quality assessment

In the quality assessments, all studies presented at least one ‘No’ answer, which suggests that, overall, there was poor reporting or methodological quality. The main questions with ‘No’ answers were in relation to the following: the sampling method, since most studies used convenience samples; the sample size, due to non-reporting of a target; the description of the subjects and setting, due to absence of information on ethnicity, comorbidities, BMI, educational level or per capita income; and lack of appropriate statistical analysis, e.g. not taking into account the number of participants with events or the total number of participants observed).

The response rate was considered unclear with regard to most studies assessing vitamin E and, consequently, no reliable estimate of vitamin E deficiency could be made. Considering that an adequate sample size depends on an estimate of prevalence, the estimated prevalence will directly influence the adequate response rate. For instance, if a prevalence of vitamin E deficiency of up to 6% is assumed, most of the studies achieved an adequate response rate. However, if a prevalence of at least 17% is assumed, none of the studies included presented an adequate response rate.

The questions for which all the answers were ‘Yes’ were in relation to the sample frame and validity of methods used for identifying the deficiencies. A detailed assessment of the methodological quality of the studies included is presented in Table 2.

**Table 2.** Methodological and reporting quality assessment, using Joanna Briggs Institute tool for prevalence studies

Studies	Questions								
	1	2	3	4	5	6	7	8	9
Madruga de Oliveira A et al.^11^^,^^32^	Yes	No ^a^	No ^c^	No ^d^	Unclear ^d^	Yes	NA ^e^	No ^f^	No ^g^
Machado et al.^12^	Yes	No ^a^	No ^c^	No ^d^	Unclear ^d^	Yes	NA ^e^	Yes	No ^g^
de Azeredo and Trugo.^14^	Yes	No ^a^	No ^c^	Yes	Yes	Yes	NA ^e^	No ^f^	Unclear ^h^
Clemente et al.^31^	Yes	No ^a^	No ^c^	No ^d^	Unclear ^d^	Yes	NA ^e^	Yes	Unclear ^h^
de Lira et al.^37^^,^^39^	Yes	No ^a^	No ^c^	No ^d^	Unclear ^d^	Yes	NA ^e^	No ^f^	Unclear ^h^
Garcia et al.^38^	Yes	No ^a^	No ^c^	No ^d^	Unclear ^d^	Yes	NA ^e^	Yes	Unclear ^h^
Gurgel et al.^29^	Yes	No ^b^	Yes	No ^d^	Unclear ^d^	Yes	NA ^e^	Yes	Unclear ^h^
Monteiro et al.^30^	Yes	No ^a^	No ^c^	No ^d^	Unclear ^d^	Yes	NA ^e^	Yes	Unclear ^h^
da Silva Ribeiro et al.^33^	Yes	No ^a^	No ^c^	No ^d^	Unclear ^d^	Yes	NA ^e^	No ^f^	Unclear ^h^
Ribeiro et al.^34^	Yes	No ^a^	No ^c^	No ^d^	Unclear ^d^	Yes	NA ^e^	Yes	Unclear ^h^
Rodrigues.^36^	Yes	No ^a^	No ^c^	Yes	Yes	Yes	NA ^e^	No ^f^	Unclear ^h^
da Silva et al.^35^	Yes	No ^a^	No ^c^	No ^d^	Unclear ^d^	Yes	NA ^e^	No ^f^	Unclear ^h^

1. Was the sample frame appropriate to address the target population? 2. Were study participants recruited in an appropriate way? 3. Was the sample size adequate? 4. Were the study subjects and setting described in detail? 5. Was data analysis conducted with sufficient coverage of the identified sample? 6. Were valid methods used for the identification of the condition? 7. Was the condition measured in a standard, reliable way for all participants? 8. Was there appropriate statistical analysis? 9. Was the response rate adequate, and if not, was the low response rate managed appropriately?

NA = not applicable; ^a^Not reported, but taken to have been convenience sampling; ^b^Reported as convenience sampling; ^c^No target sample size was reported; ^d^Most of the studies did not report ethnicity, comorbidities, body mass index, age, educational level or per capita income; ^e^Not applicable, since the methods were automated and highly replicable; ^f^Numerator (n) or denominator (N) of prevalence was not reported; ^g^These studies presented a response rate for vitamin C assessment of fewer than 320 participants; ^h^No reliable estimate of vitamin E deficiency was possible: if a prevalence of 6% or less is assumed, most of the studies achieved an adequate response rate; however, if a prevalence of 17% or higher is assumed, none of the studies presented an adequate response rate.

### Prevalence analysis

Only two studies (three papers) were found to report on the prevalence of deficiency or insufficiency of vitamin C.^11^^,^^12^^,^^32^ These studies used different cutoffs for ascorbic acid in plasma and, therefore, it was not possible to include them in any meta-analysis. De Oliveira et al.^11^^,^^32^ used a cutoff of < 22.7 mmol/l (0.4 mg/dl) and identified prevalences ranging from 27.0% (nonsmoker pregnant women) to 40.0% (smoker pregnant women), while Machado et al.^12^ found that the prevalence of vitamin C deficiency (< 11 mmol/l) was 0%, but that 12.2% of the pregnant women evaluated had suboptimal plasma levels (11-28 mmol/l).

Eleven studies reported on the prevalence of deficiency or insufficiency of vitamin E.^12^^,^^14^^,^^29^^,^^30^^,^^31^^,^^33^^,^^34^^,^^35^^,^^36^^,^^37^^,^^38^^,^^39^ Different cutoffs for alpha-tocopherol in plasma were used, but a meta-analysis with three subgroups could be conducted. Machado et al.^12^ and Monteiro et al.^30^ were not included in any vitamin E meta-analysis because their cutoffs of < 9.7 mmol/l, 9.7-16.2 mmol/l and < 7 mmol/l were not used in any other study. These two studies identified prevalences ranging from 0% to 22.4%.

Among the eleven studies, the prevalence of vitamin E deficiency ranged from 0% to 62.5%. Subgroup analyses were conducted on the mother’s weight or BMI, age, gestational weight gain, parity, delivery type, public or private maternity hospital, days after delivery, preterm or term infant, educational level, housing type (rural or urban) and per capita income. No statistical differences in prevalence (P < 0.05) were identified among these subgroups. No meta-analysis on these subgroup analyses was possible, either because only one study reported the subgroup or because different cutoffs were considered.

**Figure 1.**
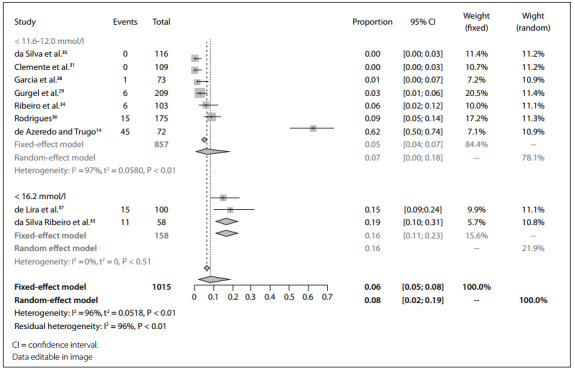
Forest plot for prevalence of vitamin E deficiency, according to alpha-tocopherol cutoff.

**Figure 2.**
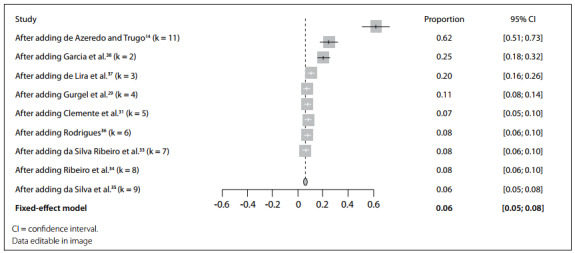
Forest plot for cumulative meta-analysis on prevalence of vitamin E deficiency, according to publication year.

In the meta-analysis for the base case, an overall prevalence of 6% (95% CI 5%-8%) was identified, while 5% (95% CI 4%-7%) and 16% (95% CI 11%-23%) were estimated for the cutoffs of 11.6-12.0 mmol/l and 16.2 mmol/l cutoffs, respectively (Figure 1). A cumulative meta-analysis was performed considering the year of publication, and this showed a smaller trend of prevalence of vitamin E deficiency or insufficiency, with a slight join point in 2015 (Figure 2).

A sensitivity analysis using the leave-one-out method was conducted but was unable to reduce the heterogeneity (89%-96%), and the overall prevalence ranged from 4% to 8% (see supplementary data: Table S3, available in OSF: https://osf.io/j9qmh/).^20^ In this analysis, withdrawal of the study by Ribeiro et al.,^34^ which was the only study that did not include breastfeeding women, resulted in a prevalence of vitamin E deficiency of 7% (95% CI 5%-8%), with I^2^ of 96%. The studies with most influence on the variations were those of de Azeredo et al.,^14^ on teenager breastfeeding, and Clemente et al.,^31^ on breastfeeding in general. Sensitivity analyses using alternative statistical methods identified prevalences ranging from 8% to 17% (see supplementary data: Table S4, available in OSF: https://osf.io/j9qmh/).^20^

**Figure 3.**
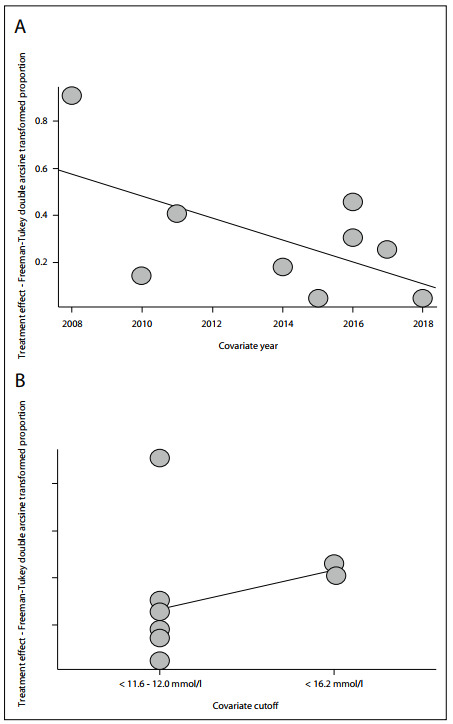
A) Meta-regression plot for publication year (vitamin E deficiency); B) Meta-regression plot for alpha-tocopherol cutoff.

Meta-regression analyses were conducted on publication year (P < 0.01) and cutoffs (P < 0.001), and both of these variables explained the heterogeneity (Figure 3). No meta-regression or subgroup analyses on other variables was possible. It was also not possible to conduct statistical and visual analyses on publication bias for any meta-analysis because the requirements for a minimum number of studies or different results and sample sizes were not met.

## DISCUSSION

In this systematic review, twelve studies assessing the prevalence of vitamins C and E deficiency, especially among breastfeeding women, were identified. Two studies reported on vitamin C deficiency (0% to 40%) and eleven reported on vitamin E deficiency (0% to 62.5%), with a mean prevalence of 6% identified through the meta-analysis. The low frequency of assessment of vitamin C and E deficiencies in the past may explain why no systematic review reporting the prevalence of deficiency of these vitamins was found.^6^ It is important to note that our findings suggest that deficiency of vitamins C and E could constitute a public health problem in Brazil, depending on the study and the cutoff for plasma alpha-tocopherol or ascorbic acid under consideration.

It is likely that breastfeeding women have lower vitamin C status due to transfer of vitamin C to the growing infant via breastmilk.^40^ Maternal intake of vitamin C in the diet but not as a supplement has been shown to determine the concentration of vitamin C in breast milk,^41^ and this was found to vary with season. Therefore, maternal ascorbic acid intake and education about healthy nutrition (consumption of vegetables and fruits) are important.

Furthermore, during pregnancy, placental transfer of vitamin E to the fetus is limited. Thus, breast milk is the only source of this nutrient for infants that are exclusively breastfed.^42^ The composition of human milk depends on the stage of breastfeeding, time of the day, time since last meal, nutrition, maternal age, gestational age of the newborn and other individual maternal factors.^43^ Therefore, it is important to diagnose deficiencies of vitamins C and E among breastfeeding women, since intake of these vitamins is an important way to supply the newborn with essential antioxidant protection and to stimulate immune system development.^43^

Indeed, insufficient evidence of deficiencies of vitamins C and E worldwide has led to a lack of definition of what constitutes adequate intake of these micronutrients. It has also led to use of different cutoffs for defining deficiency or insufficiency. As expected, in studies evaluating alpha-tocopherol (vitamin E), the higher the cutoff point was (alpha-tocopherol < 16.2 mmol/l), the greater the prevalence of deficiency also was (16%). This suggests that there is a need for studies to establish the cutoff taking into consideration the patients’ clinical and physiological conditions (e.g. pregnant women, breastfeeding women or infants). It has, for instance, been found that maternal deficiency influenced the level of vitamin E in the umbilical cord, but not in the colostrum. This suggests that strategies for solving vitamin E deficiency should consider differences among pregnant and breastfeeding women.^33^

All the studies included here showed low reporting or methodological quality, thus producing findings with low confidence, mainly due to inappropriate sampling methods and sample sizes, which consequently did not provide representative samples of the base populations. High inconsistency was also identified, which further downgrades the confidence in the prevalence rates reported. These issues are common in observational studies.^17^^,^^44^^,^^45^ This high inconsistency suggested that the studies should not be included in the same meta-analysis because of their different evaluation methods (e.g. the methods used to assess deficiency) or participant characteristics (e.g. age, ethnicity, educational level, per capita income or sociodemographic factors). In fact, most of the studies did not report the characteristics of the participants, which made it impossible to conduct robust analyses for exploring the heterogeneity, or to identify possible factors associated with deficiency or insufficiency of vitamins C and E.

Several options for vitamin supplements containing vitamins C and E are available in Brazil, and some of them are included in the Brazilian National List of Essential Medicines (RENAME).^46^ Distribution of sachets containing a mixture of micronutrients forms part of the Brazilian strategy for strengthening baby feeding with micronutrients in powder (NutriSUS).^47^ Moreover, policies for iron + folic acid^48^ and vitamin A^49^ supplementation for pregnant and breastfeeding women exist. Nonetheless, no national policy regarding supplementation with any other vitamin (e.g. C and E) exists.

On the other hand, little is known regarding the benefits of use of these supplements among pregnant women. A Cochrane systematic review found that routine supplementation with vitamin E in combination with other supplements, to prevent fetal death, neonatal death, premature birth, preeclampsia, premature rupture of membranes (at term or preterm) or fetal growth restriction, was not supported by the current data.^6^ Another systematic review, which assessed interventions consisting of vitamin C supplementation alone or in combination with other supplements, found that there was no reduction in prevention of fetal or neonatal death, poor fetal growth, preterm birth or pre-eclampsia.^50^ Therefore, it is important to assess the risks relating to deficiency of vitamins C and E, as well as the benefits from supplementation of these vitamins.

The risk of hypervitaminosis^51^ or supplement-drug interactions has been documented.^52^^,^^53^ Drug interactions with vitamins can be of particular importance and are well documented. These include the following situations: beta-blockers may present reduced absorption if used concurrently with vitamin C supplementation; mineral oil and antacids containing aluminum hydroxide can reduce the absorption of fat-soluble vitamins; vitamin C supplementation can inhibit the action of some antibiotics; use of proton pump inhibitors can cause vitamin C deficiency; and excessive vitamin E supplementation can reduce the absorption of vitamins A and K.^52^^,^^53^ Therefore, care should be taken with regard to irrational use of supplements through self-medication or prescription. Indeed, robust evidence to assess the problem of vitamin deficiencies and their outcomes among women is needed before establishing potential strategies.

One limitation of this study, like in any systematic review, was that some studies may have been missed. To overcome this limitation, extensive investigation of the gray literature and manual searches to find unpublished studies were conducted, which found several studies that had not been retrieved through electronic searches. This high number of studies identified through manual search might be seen as a limitation of the search strategy. However, one hypothesis for explaining this occurrence is that the titles and abstracts of many studies may have been inadequately drafted in relation to the study subject or may not have been correctly indexed, which might have hindered retrieval. Lastly, another limitation was the absence of robust analysis on the potential factors associated with vitamin C and E deficiencies, due to poor reporting and the small size of the meta-analyses (studies and participants).

## CONCLUSION

Although the studies identified in this systematic review showed poor reporting and poor methodological quality, the current evidence suggests that a mild-to-moderate problem exists regarding the prevalence of deficiencies of vitamin C (ranging from 0% to 40%) and vitamin E (5% to 16%), especially among breastfeeding women and in studies in Rio Grande do Norte. Thus, it seems that designing and implementing policies to address this problem is not seen as a national priority because the deficiency problem among women of childbearing age in Brazil is not accurately represented. Future studies should consider using standard cutoffs for plasma alpha-tocopherol and ascorbic acid, random probabilistic sampling, appropriate sample sizes and predefined subgroup analyses, in order to adequately inform the prevalences of deficiencies of vitamins C and E and associated factors among women of childbearing age (non-pregnant, pregnant and breastfeeding women), and to support potential healthcare policies.
